# An extended data mining method for identifying differentially expressed assay-specific signatures in functional genomic studies

**DOI:** 10.1186/1756-0381-3-11

**Published:** 2010-12-17

**Authors:** Derrick K Rollins, AiLing Teh

**Affiliations:** 1 Department of Chemical and Biological Engineering, Iowa State University, Ames, IA 50011, USA; 2 Department of Statistics, Iowa State University, Ames, IA 50011, USA

## Abstract

**Background:**

Microarray data sets provide relative expression levels for thousands of genes for a small number, in comparison, of different experimental conditions called *assays*. Data mining techniques are used to extract specific information of genes as they relate to the assays. The multivariate statistical technique of principal component analysis (PCA) has proven useful in providing effective data mining methods. This article extends the PCA approach of Rollins et al. to the development of ranking genes of microarray data sets that ***express most differently ***between two biologically different grouping of assays. This method is evaluated on real and simulated data and compared to a current approach on the basis of false discovery rate (FDR) and statistical power (SP) which is the ability to correctly identify important genes.

**Results:**

This work developed and evaluated two new test statistics based on PCA and compared them to a popular method that is not PCA based. Both test statistics were found to be effective as evaluated in three case studies: (i) exposing *E. coli *cells to two different ethanol levels; (ii) application of myostatin to two groups of mice; and (iii) a simulated data study derived from the properties of (ii). The proposed method (PM) effectively identified critical genes in these studies based on comparison with the current method (CM). The simulation study supports higher identification accuracy for PM over CM for both proposed test statistics when the gene variance is constant and for one of the test statistics when the gene variance is non-constant.

**Conclusions:**

PM compares quite favorably to CM in terms of lower FDR and much higher SP. Thus, PM can be quite effective in producing accurate signatures from large microarray data sets for differential expression between assays groups identified in a preliminary step of the PCA procedure and is, therefore, recommended for use in these applications.

## Introduction

It is well known that living organisms have complicated gene structures. However, while major advancements have been made in recent years, understanding of the biological functions of each individual gene is still quite limited. Active research is strongly focused on understanding the behavior of genes and as well as the highly complex metabolism and regulatory network inside living cells [1]. This effort falls under a molecular biological field called functional genomics (FG). There are at least three areas in which experimental techniques are widely applied in FG: transcriptomics, proteomics, and metabolomics [2]. A combination of leading scientific techniques as well as powerful mathematical and statistical tools for data analysis makes the task of identifying important transcriptome, proteome, and metabolome corresponding to a biological effect promising. Typical studies in these areas involve the identification of possible behavior and responses of species under various genetic backgrounds as well as environmental factors (i.e. assay).

There are different high technology techniques applied in FG field to advance understanding of the transcriptional genetic response of many organisms in various environmental perturbations [1]. One of the techniques that have been adopted in this field is a multiplex technology called DNA microarray [3]. A new technique that is becoming popular and will probably displace array-based measurement in FG is next-generation sequencing (RNAseq) [4,5]. These techniques have the ability to generate data sets that consist of expression levels of thousands of genes, providing a wealth of information that is hidden by high noise levels, low signal levels, and a relatively small number of experimental units to the number of genes studied. More specifically, since the data set containing the gene expression measurements consists of a lot more genes than assays, analytical techniques are needed to provide accurate gene identification under a large number of gene candidates that is much greater than the number of experimental runs.

To achieve this objective, traditional statistical methods, such as principal component analysis (PCA) [2-8], the focus of this article, are being retrofitted to provide effective statistical inference in this challenging context of microarray data analysis. Other methods used in this field included linear model analysis [9-14], Bayesian method [15-17] and network component analysis (NCA) [18-20]. Thus, statistics is playing a critical role through the development of methodologies that give high statistical power (SP) (i.e., accurate identification), and low false discovery rate (FDR)[21] (i.e. low misidentification). To this end, this article introduces two new PCA based statistics for determining gene rank for *differential expression *between two PCA identified assay groups. This work extends the technique introduced by Rollins et al.[2] that determines gene rank for a *single *PCA identified assay group. Thus, the proposed method (PM) in this work is aimed at finding the genes with high expression levels in one group and low expression levels in the other group.

The PM uses PCA to first establish the existence of the assay groupings of interest. Then using the results that established the grouping, the differential contribution for each gene is determined using a statistic based on eigenvalues. This article proposes and evaluates two statistics. The first one is the group averaged difference of eigenvalue linear combinations that we call *T_diff_*. The second one divides *T_diff _*by its estimated pooled standard deviation that we call *T_scaled_*. The genes are ranked based on the largest absolute value of these statistics. The PM is evaluated against the ranking determined by the well known Student's *t*-statistic [14] that we call *T_pooled _*in this work. We will refer to *T_pooled _*as the current method (CM) which is actually a subclass of the PM that weighs each assay equally in each group. Note that for the CM the assay members in each group is not established based on the data but by á priori considerations. In contrast, for the PM the data drives the assay weight as well as group assignment of the assays.

The CM and PM are applied in the following three case studies to compare their effectiveness (i.e., power) in identifying assay-specific signature: (i) exposure of *E. coli *cells to two different levels of ethanol concentration [22]; (ii) the use of myostatin as inhibitor of skeletal muscle growth for five 5-weeks-old myostatin and non-treated mice [10]; and (iii) a simulation study based on statistical properties of the second case study.

This work is organized into the following sections. The Background Section gives a brief review of PCA and connects it to our application in FG's data analysis. This section is followed by the Methods Section that derives and presents the test statistics of the CM and PM. These test statistics are evaluated and compared in three studies in the Results and Discussion Section. The final section summarizes the results and gives concluding remarks on the contribution of this work.

## Background

The microarray data set is given as an *m *by *n *matrix **X **where *n *is the number of assays expressed along columns (i.e. variables) and *m *represents the number of genes expressed along rows. The cells in this matrix are given as *x_ij _*which is the expression level of the *i*^th ^gene for the *j*^th ^assay (i.e. condition). Principal component analysis (PCA) is a multivariate technique that mathematically transforms (rotates) the original coordinate system to a new orthogonal coordinate system based on correlations among the variables [23]. The principal components (PCs) are eigenvectors generated from either the covariance matrix (scaled sum of squares and cross products) or the correlation matrix (sums of squares and cross products from standardized data) of the variables involved. They are used to construct *n *linear combinations of the *n *variables that can be thought of as *n *pseudo variables [23]. A PC is rank ordered by the amount of variation in the original data set that it captures.

**Figure 1:**
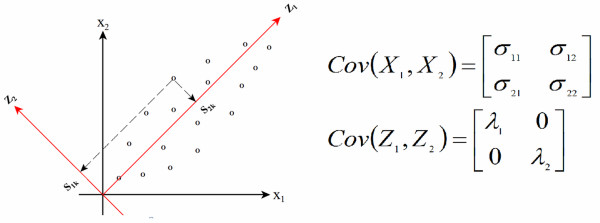
**Visual representation of the original data system and the rotated data system**. The figure represents the original data system on the horizontal and vertical axis while the new rotated data system is represented as z_1 _and z_2_. Variance-covariance matrices for original data system and rotated data system are shown on the right of the figure.

An illustration is given in Figure 1 that shows a visual representation of a two-dimensional data system (*x*) and a rotated data system (*z*). As shown, the new coordinated system points *z_1 _*in the direction with the greatest spread in the data. The other variable, *z_2_*, points in a direction that is orthogonal to *z_1_*, but also seeks to maximize spread in this direction. The first PC determines *z_1 _*and the second PC determines *z_2_*. A data matrix of rank *n *will give *n *PCs that are linear combinations of the variables in the original data matrix that can be described as *n pseudo *variables. The goal in this application of PCA is to obtain at least one pseudo variable that represent the biological behavior of interest. This can be a PC that represents a small portion of the total variation making it a potentially very powerful data mining approach.

**Figure 2:**
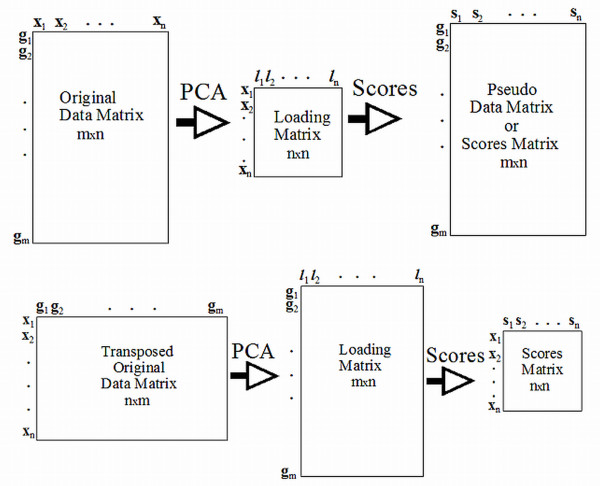
**Visual representation of data, loading, and score matrices for *X *and *X^T^***. The upper part of this figure represents the EG procedure while the bottom part of this figure represents the EA procedure. Note that dimension of the data matrix used for EG and EA and different.

The top of Figure 2 shows the relationship between the original data matrix, **X**, the *n *by *n *PC loading matrix, **L**, and the *m *by *n *pseudo data matrix, called the scores matrix, **S**. The PCs derived from **X **are called eigengenes (EG) because the elements of **S **represent pseudo values for gene expression. In Figure 2 the bottom set of matrices are derived from the transpose of **X **which is an *n *by *m *matrix. In this case the loading matrix is *m *by *n *in dimension and the scores matrix is *n *by *n *in dimension. The PCs derived from the transpose of **X **are called eigenassays (EA) because the elements of the scores matrix represent pseudo assays. The proposed method (PM), following Rollins et al. [2], uses both EG and EA approaches to develop signatures sets of ranked genes. In the next section we derive the EG and EA statistics for determining gene contribution for the PM.

## Methods

### Eigengene Contribution Approach

The first step in the eigengene (EG) approach of the PM is to standardized the elements of **X **to give the standardized matrix **Z **with each element equal to $z_{ij}=\left(x_{ij}-{\bar{x}}_{j}\right)/s_{j}$, where ${\bar{x}}_{j}$ and *s_j _*are the sample mean and sample standard deviation of the data in column *j*, respectively. The following distributional assumptions are made for simplicity and are taken as the scope of this work: $x_{ij}\overset{indep}{\sim }N\left(\mu_{x_{j}},\sigma_{x_{i}}^{2}\right),\;z_{ij}\sim N\left(\mu_{z_{j}},\sigma_{z_{i}}^{2}\right),\;{\bar{x}}_{j}\overset{indep}{\sim }N\left(\mu_{x_{j}},\sigma_{j}^{2}/m\right),\text{and}\;\text{E}\left[s_{j}^{2}\right]=\sigma_{j}^{2}\;\forall \;j$. These assumptions indicate that each assay can have it own mean expression level, $\mu_{x_{j}}\left(j=1,\ldots ,n\right)$, and that the variance of each gene is constant across assays but can be different for different genes. Also, $\mu_{z_{j}}=0\;\;\forall i,j\;\text{since}\;\text{E}\left[x_{ij}\right]=\text{E}\left[{\bar{x}}_{j}\right]=\mu_{x_{j}}=\forall i,j$. These assumptions will be utilized later after proposing the test statistics. The elements of the EG scores matrix, **S**^EG^, are determined by

(1) $$\begin{array}{c} s_{ij}^{EG}=\ell_{1j}^{EG}z_{i1}+\ell_{2j}^{EG}z_{i2}+\ldots +\ell_{nj}^{EG}z_{in}=\sum_{k=1}^{n}\ell_{kj}^{EG}z_{ik}\\ =g_{ij1}^{EG}+g_{ij2}^{EG}+\ldots +g_{ijn}^{EG}=\sum_{k=1}^{n}g_{ijk}^{EG};\\ i=1,\ldots ,m;j=1,\ldots ,n;k=1,\ldots ,n \end{array}$$

where $s_{ij}^{EG}$ is the score for the *i*^th ^gene using the *j*^th ^vector of EG loadings, $\ell_{ij}^{EG}$ is the *i*^th ^loading for the *j*^th ^EG vector, and $g_{ijk}^{EG}$ is the contribution for the *i*^th ^gene, on the *k*^th ^assay from the *j*^th ^EG loading vector. Let A = Group A with *n_A _*assay members and B = Group B with *n_B _*assay members with no members in common with Group A such that

(2) $$\text{2}\leq n_{A}+n_{B}\leq n$$

The mean contribution for *i*^th ^gene from the *j*^th ^EG loading vector for Groups A and B, respectively are

(3) $${\bar{g}}_{ij}^{EG_{A}}=\frac{1}{n_{A}}\sum_{over\;k\prime }\ell_{k\prime j}^{EG}z_{ik\prime }=\frac{1}{n_{A}}\sum_{over\;k\prime }g_{ijk\prime }^{EG}$$

(4) $${\bar{g}}_{ij}^{EG_{B}}=\frac{1}{n_{B}}\sum_{over\;k\prime \prime }\ell_{k\prime \prime j}^{EG}z_{ik\prime \prime }=\frac{1}{n_{B}}\sum_{over\;k\prime \prime }g_{ijk\prime \prime }^{EG}$$

where *k' *and *k'' *are the assay members in Groups A and B, respectively. Finally, the EG differential gene contribution between Groups A and B for the *i*^th ^gene from the *j*^th ^EG loading vector is given as

(5) $$d\;{\bar{g}}_{ij}^{EG}={\bar{g}}_{ij}^{EG_{A}}-{\bar{g}}_{ij}^{EG_{B}}$$

The basic difference between the method in Rollins et al. [2] and this extension is that work developed gene signatures for individual groups using equations of the form given by (3) and (4) and this work uses equation of the form given by Eq. 5.

### Eigenassay Contribution Approach

As stated above, the EA approach uses the transpose of **X **as the data matrix treating the genes as the variables. Following Rollins et al. [2], **X**^T ^is not standardized in the EA approach as in the EG approach. The elements of scores matrix, **S**^EA^, are determined from Eq. 6 as follows:

(6) $$\begin{array}{c} s_{ij}^{EA}=\ell_{1j}^{EA}x_{1i}+\ell_{2j}^{EA}x_{2i}+\ldots +\ell_{mj}^{EA}x_{mi}\\ =\sum_{p=1}^{m}\ell_{pj}^{EA}x_{pi}=\sum_{p=1}^{m}g_{ijp}^{EA};\\ i=1,\ldots ,n;j=1,\ldots ,n;p=1,\ldots ,m \end{array}$$

where $s_{ij}^{EG}$ is the score for the *i*^th ^assay using the *j*^th ^vector of EA loadings, $\ell_{ij}^{EG}$ is the *i*^th ^loading for the *j*^th ^EA vector, and $g_{ijp}^{EA}$ is the contribution for the *p*^th ^gene, on the *i*^th ^assay from the *j*^th ^EA loading vector. As above, for A = Group A with *n_A _*assay members and B = Group B with *n_B _*assay members with no members in common with Group A, we obtain the contribution expressions as follows. The mean contribution for *p*^th ^gene from the *j*^th ^EA loading vector for Groups A and B are

(7) $${\bar{g}}_{jp}^{EA_{A}}=\frac{\ell_{pj}^{EA}}{n_{A}}\sum_{over\;i\prime }x_{pi\prime }=\frac{1}{n_{A}}\sum_{over\;i\prime }g_{i\prime jp}^{EA}$$

(8) $${\bar{g}}_{jp}^{EA_{B}}=\frac{\ell_{pj}^{EA}}{n_{B}}\sum_{over\;i\prime \prime }x_{pi\prime \prime }=\frac{1}{n_{B}}\sum_{over\;i\prime \prime }g_{i\prime \prime jp}^{EA}$$

respectively, where *i' *and *i" *represent the assay members in Groups A and B, respectively. Finally, the EA differential gene contribution between Groups A and B for the *p*^th ^gene from the *j*^th ^EG loading vector is given as

(9) $$d\;{\bar{g}}_{jp}^{EA}={\bar{g}}_{jp}^{EA_{A}}-{\bar{g}}_{jp}^{EA_{B}}$$

### Test Statistics

The next step after deriving the gene contribution equations is to define the decision or test statistics based on these derivations. *T_diff _*for EG and EA are equivalent to Eqs. 5 and 9, respectively. More specifically,

(10) $$\begin{array}{c} T_{diff_{ij}}^{EG}=d\;{\bar{g}}_{ij}^{EG}={\bar{g}}_{ij}^{EG_{A}}-{\bar{g}}_{ij}^{EG_{B}}\\ =\frac{1}{n_{A}}\sum_{over\;k\prime }\ell_{k\prime \;j}^{EG}z_{ik\prime }-\frac{1}{n_{B}}\sum_{over\;k\prime \prime }\ell_{k\prime \prime j}^{EG}z_{ik\prime \prime } \end{array}$$

(11) $$\begin{array}{l} T_{diff_{jp}}^{EA}=d\;{\bar{g}}_{jp}^{EA}={\bar{g}}_{jp}^{EG_{A}}-{\bar{g}}_{jp}^{EG_{B}}\\ =\frac{\ell_{pj}^{EA}}{n_{A}}\sum_{over\;i׳}x_{pi׳}-\frac{\ell_{pj}^{EA}}{n_{B}}\sum_{over\;i"}x_{pi"}\\ =\ell_{pj}^{EA}\left({\bar{x}}_{Ap}-{\bar{x}}_{Bp}\right) \end{array}$$

The variances for the components of these equations are given below by treating the loadings as fixed variables (making these expressions approximations):

(12) $$\begin{array}{c} V\left({\bar{g}}_{ij}^{EG_{A}}\right)=V\left(\frac{1}{n_{A}}\sum_{over\;k'}\ell_{k'j}^{EG}z_{ik'}\right)\\ \approx \frac{1}{n_{A}^{2}}\sum_{over\;k'}{\left(\ell_{k'j}^{EG}\right)}^{2}\sigma_{z_{i}}^{2}=\frac{\sigma_{zi}^{2}}{n_{A}^{2}}\sum_{over\;k'}{\left(\ell_{k'j}^{EG}\right)}^{2} \end{array}$$

(13) $$\begin{array}{c} V({\bar{g}}_{ij}^{EG_{B}})=V\left(\frac{1}{n_{B}}\sum_{over\;k''}\ell_{k"j}^{EG}z_{ik"}\right)\\ \approx \frac{1}{n_{B}^{2}}\sum_{over\;k"}{\left(\ell_{k"j}^{EG}\right)}^{2}\sigma_{z_{i}}^{2}=\frac{\sigma_{z_{i}}^{2}}{n_{B}^{2}}\sum_{over\;k'}{\left(\ell_{k"j}^{EG}\right)}^{2} \end{array}$$

(14) $$\begin{array}{c} V({\bar{g}}_{jp}^{EA_{A}})=V\left(\frac{\ell_{pj}^{EA}}{n_{A}}\sum_{over\;i'}x_{pi'}\right)\\ \approx \frac{{\left(\ell_{pj}^{EA}\right)}^{2}}{n_{A}^{2}}n_{A}\sigma_{xp}^{2}={\left(\ell_{pj}^{EA}\right)}^{2}\frac{\sigma_{xp}^{2}}{n_{A}} \end{array}$$

(15) $$\begin{array}{c} V({\bar{g}}_{jp}^{EA_{B}})=V\left(\frac{\ell_{pj}^{EA}}{n_{B}}\sum_{over\;i"}x_{pi"}\right)\\ \approx \frac{{\left(\ell_{pj}^{EA}\right)}^{2}}{n_{B}^{2}}n_{B}\sigma_{xp}^{2}={\left(\ell_{pj}^{EA}\right)}^{2}\frac{\sigma_{xp}^{2}}{n_{B}} \end{array}$$

Thus, combining Eqs. 10-11, the variances for $T_{diff_{ij}}^{EG}$ and $T_{diff_{jp}}^{EA}$ respectively are:

(16) $$\begin{array}{c} V(T_{diff_{ij}}^{EG})\approx \frac{\sigma_{z_{i}}^{2}}{n_{A}^{2}}\sum_{o\nu er\;k'}{\left(\ell_{k'\;j}^{EG}\right)}^{2}+\frac{\sigma_{z_{i}}^{2}}{n_{B}^{2}}\sum_{over\;k"}{\left(\ell_{k''\;j}^{EG}\right)}^{2}\\ =\sigma_{z_{i}}^{2}\left[\frac{1}{n_{A}^{2}}\sum_{over\;k'}{\left(\ell_{k'\;j}^{EG}\right)}^{2}+\frac{1}{n_{B}^{2}}\sum_{over\;k"}{\left(\ell_{k"\;j}^{EG}\right)}^{2}\right] \end{array}$$

(17) $$\begin{array}{c} V(T_{diff_{pj}}^{EA})\approx {\left(\ell_{pj}^{EA}\right)}^{2}\frac{\sigma_{xp}^{2}}{n_{A}}+{\left(\ell_{pj}^{EA}\right)}^{2}\frac{\sigma_{xp}^{2}}{n_{B}}\\ ={\left(\ell_{pj}^{EA}\right)}^{2}\sigma_{xp}^{2}\left[\frac{1}{n_{A}}+\frac{1}{n_{B}}\right] \end{array}$$

The scale test statistic in the EG case can now be given by dividing Eq. 10 by the estimated standard deviation using Eq. 16:

(18) $$\begin{array}{c} T_{scaled_{ij}}^{EG}=\frac{T_{diff_{ij}}^{EG}}{{\left[\hat{V}\left(T_{diff_{ij}}^{EG}\right)\right]}^{}}\\ =\frac{\frac{1}{n_{A}}\sum_{over\;k'}\ell_{k'\;j}^{EG}z_{ik'}-\frac{1}{n_{B}}\sum_{over\;k"}\ell_{k"\;j}^{EG}z_{ik"}}{s_{pooled\;z_{i}}\sqrt{\frac{1}{n_{A}^{2}}{\sum_{over\;k'}\left(\ell_{k'\;j}^{EG}\right)}^{2}+\frac{1}{n_{B}^{2}}{\sum_{over\;k"}\left(\ell_{k"\;j}^{EG}\right)}^{2}}} \end{array}$$

where

(19) $$s_{pooled\;z_{i}}^{2}=\frac{n_{A}-1}{n_{A}+n_{B}-2}s_{Az_{i}}^{2}+\frac{n_{B}-1}{n_{A}+n_{B}-2}s_{Bz_{i}}^{2}$$

$s_{Az_{i}}^{2}$ and $s_{Bz_{i}}^{2}$ are the sample variances for the standardized expression levels for Groups A and B, respectively, corresponding to the *i*^th ^gene. Note that when $x_{ij}\overset{indep}{\sim }N\left(\mu_{x_{j}},\sigma^{2}\right),\;\forall i,j,\;\text{then}\;{\bar{x}}_{j}\overset{indep}{\sim }N\left(\mu_{x_{j}},\sigma^{2}/m\right)\forall \;j$. Therefore, $Z_{ij}\;=\left(x_{ij}\;-{\bar{x}}_{j}\right)/s_{j}∼N\left(0,1\right),$ approximately, since ${\bar{x}}_{j}\approx \mu_{x_{j}}$ and $s_{j}^{2}\approx \sigma^{2}\forall \;j$ because *m *is very large. In this case where the variation of the assays are all similar, V(*z_ij_*) is taken to equal 1 and

(20) $$T_{scaled_{ij}}^{EG}=\frac{\frac{1}{n_{A}}\sum_{over\;k'}\ell_{k'\;j}^{EG}z_{ik'}-\frac{1}{n_{B}}\sum_{over\;k"}\ell_{k"\;j}^{EG}z_{ik"}}{\sqrt{\frac{1}{n_{A}^{2}}{\sum_{over\;k'}\left(\ell_{k'\;j}^{EG}\right)}^{2}+\frac{1}{n_{B}^{2}}\sum_{over\;k"}{\left(\ell_{k"\;j}^{EG}\right)}^{2}}}$$

Similarly, the scaled test statistic in the EA case can also be given now by dividing Eq. 11 by the estimated standard deviation using Eq. 17:

(21) $$\begin{array}{c} T_{scaled_{jp}}^{EG}=\frac{T_{diff_{jp}}^{EA}}{{\left[\hat{V}\left(T_{diff_{jp}}^{EA}\right)\right]}^{1/2}}\\ =\frac{\ell_{pj}^{EA}\left[\frac{1}{n_{A}}\sum_{over\;i'}x_{pi'}-\frac{1}{n_{B}}\sum_{over\;i"}x_{pi"}\right]}{\ell_{pj}^{EA}s_{pooled_{x_{p}}}\sqrt{\left[\frac{1}{n_{A}}+\frac{1}{n_{B}}\right]}}\\ =\frac{{\bar{x}}_{A_{p}}-{\bar{x}}_{B_{p}}}{s_{pooled_{x_{p}}}\sqrt{\left[\frac{1}{n_{A}}+\frac{1}{n_{B}}\right]}} \end{array}$$

where

(22) $$s_{pooled\;x_{p}}^{2}=\frac{n_{A}-1}{n_{A}+n_{B}-2}s_{Ax_{p}}^{2}+\frac{n_{B}-1}{n_{A}+n_{B}-2}s_{Bx_{p}}^{2}$$

$s_{Ax_{p}}^{2}$ and $s_{Bx_{p}}^{2}$ are the sample variances for the un-standardized expression levels for Groups A and B, respectively, corresponding to the *p*^th ^gene. Note that $T_{scale_{JP}}^{EA}$ is independent PCA loadings and thus, does not benefit from PCA. In actuality, Eq. 21 is the commonly known Student's *t-*statistics [14]; thus,

(23) $$T_{pooled,p}=T_{scale_{JP}}^{EA}$$

From Eq. 23 it is clear that scaling the EA differential contribution is not providing any new technique in PCA and therefore is not a useful result under the PM. Thus, we do not propose scaling for the EA approach.

The steps for applying the PM are as follows:

1. Standardize **X **to obtain **Z**.

2. Obtain the loading and scores matrices for **X **(EG) based on correlation.

3. Obtain the loading and scores matrices for **X***^T ^*(EA) based on covariance.

4. For each of the *n *EG loading vectors, plot its loadings against the assay number. Select the plot(s) that separate the assays into desired or interesting groups for further analysis.

5. For each *n *EA score vectors, plot its scores against the assay number. Select the plot(s) that separate the assays into desired or interesting groups for further analysis.

6. For each selected EG loading vector in Step 4, using **Z **and Eq. 5 determine the differential EG contribution for each gene.

7. For each selected EA loading vector in Step 5, using **X **and Eq. 9 determine the differential EA contribution for each gene.

8. For each case in Steps 6 and 7, rank order the differential contribution and then table (with the corresponding gene) and plot these values against the rank. These signature plots can be used to determine where to make cutoffs as described in Rollins et al. [2].

In the next section we evaluate the proposed test statistics that we have derived in this section against a current method that uses the Student's *t*-test statistic. This work also includes an evaluation to determine when it is better to choose *T_diff _*or *T_scale_*.

## Results and Discussion

The best choice for a test statistics is the one that has the highest statistical power (SP) and the lowest false discovery rate (FDR) [21]. This section presents three case studies to evaluate the proposed test statistics against one another and against a current method (CM) that uses *T_pooled_*. The first study revisits the single group analysis in Rollins et al. [2] involving exposure of *E. coli *cells to two different levels of ethanol concentration [2,22]. The second study applies the proposed method (PM) to data from Steelman et al. [10]. This data set involves the use of myostatin as an inhibitor of skeletal muscle growth for five 5-weeks-old myostatin (called "mutant") and non-treated (called "wild-type") mice in each group. The third study is a mathematically simulated data study using characteristics of the data from Study 2.

### Exposure of *E. coli *cells Study

The data set for the first case study contains *E. coli *cells that were exposed to two different ethanol concentrations. In Rollins et al.[2] ranked signatures were obtained for non-ethanol (i.e., non-treated) (Group A) and ethanol (Group B) separately. Thus, these signatures ranked the genes based on their contribution to the score of their group. However, the goal of this work is to obtain a ranked signature of the genes that is based on the ***difference of gene contribution ***between the two groups. Therefore, under this objective, genes with high contribution in both groups would not be ranked high; whereas, genes with low contribution in one group and high contribution in the other group could be ranked high based on the greatest negative, positive, or absolute difference, depending on the interests of the experimenter. For this study, we ranked the genes based on absolute difference for evaluative purposes.

**Figure 3:**
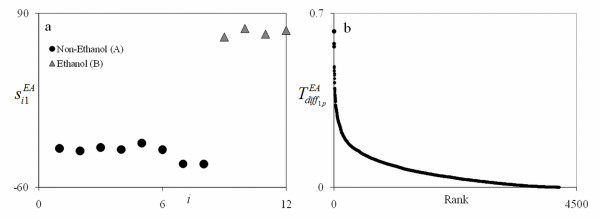
**EA1 Score plot (a) and gene signature plot (b) for the *E. coli *in ethanol and non-ethanol study**. The score plot shows excellent separation for the non-ethanol group (A) and ethanol group (B). For the signature plot on the right, the rank decreases as the number increases. The top ranking genes seen on the upper left in plot (b) shows distinct separation as an evidence of the vertical line gap between the genes.

The results of this study using the PM are given in Table 1 and Figure 3. These results were obtained from the first PC for an EA analysis (since it indicated the strongest separation) using $T_{diff_{i1}}^{EA}$ only to determine differential gene contribution. This PC was selected, as supported by Figure 3, because it separated the two groups in the score plot quite well. The plot on the right in Figure 3 gives the differential contribution calculated from $T_{diff_{i1}}^{EA}$ by rank with the rank decreasing with increasing value on the horizontal axis. As this figure shows, the top genes clearly standout by their distinct separation and how they line up almost vertically along the vertical axis. Table 1 gives the top 20 genes that expressed the most differently between ethanol treated and non-ethanol treated groups. This list contains some of the top genes in the ethanol and non-ethanol signatures in Rollins et al.[2] as indicated. In addition, it contains genes that were not ranked very high in either signature. However, note that each gene is at opposite ends of the signatures in Rollins et al.[2] in support of their differential significance. Thus, the PM has potentially found genes that might express relatively low within assays of similar conditions but quite differently between assays of different conditions. Follow up experiments would be necessary to verify these findings which are beyond the scope of this work.

**Table 1:** Top 20 genes that showed distinct difference between ethanol and non-ethanol along with their ranking

Rank	Gene Name	EtOH Rank*	Non-EtOH Rank*	Rank	Gene Name	EtOH Rank*	Non-EtOH Rank*
1	*b2387*	729	2001	11	*argT*	925	2330
2	*ybdO*	558	2182	12	*argH*	2	4286
3	*b1455*	959	2120	13	*ycbE*	317	3626
4	*gltD*	2884	151	14	*b0538*	328	2658
5	*appY*	360	2457	15	*citB*	372	2642
6	*caiA*	5	3810	16	*wbbH*	2952	408
7	*b0960*	2664	787	17	*ccmD*	2605	1083
8	*yaiD*	1	4284	18	*agaA*	885	2483
9	*b1815*	3178	43	19	*ymcC*	568	2587
10	*ydaK*	375	2548	20	*abc*	389	2705

The "*" gives the rank in the ethanol and non-ethanol signature in Rollins et al. [2].

### Skeletal Muscle Growth in Mice Study

The second study is a data set that involved the use of myostatin as inhibitor of skeletal muscle growth for five 5-week-old myostatin (called "mutant") and non-treated (called "wild-type") mice in each group. A powerful method for ranking genes and determining the size of signatures is the Q-method developed by Storey and Tibshirani [12]. The Q-method uses *T_pooled _*and a novel method for achieving high SP and low FDR. The Q-method first uses *T_pooled _*to obtain p-values then convert to q-values to determine where to cut-off signatures based on a maximum q-value. Given that the q-value is related to the p-value, one could also rank genes based on p-values or their *T_pooled _*values which are inversely related. Since we are primarily interested in ranking genes in this work, we will compare the techniques based on the abilities of *T_pooled _*and the PM to find top ranked genes.

**Figure 4:**
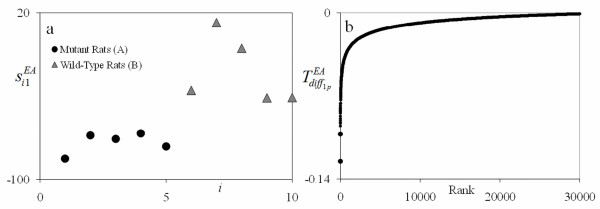
**EA1 Score plot (a) and gene signature plot (b) for the skeletal muscle growth in mice study**. The score plot shows excellent grouping for the mutant mice (A) and the wild-type mice (B) assays. For the signature plot on the right, the rank decreases as the number increases. The top ranking genes seen in plot (b) show distinct separation by how they line up almost vertically along the vertical axis

PCA results for PM are given in Figure 4. These results were obtained from the first PC for an EA analysis (since it indicated the strongest separation) using $T_{diff_{i1}}^{EA}$ only to determine differential gene contribution. This PC was selected, as supported by Figure 4, because it separated the two groups in the score plot quite well. As shown by the $T_{diff_{i1}}^{EA}$ plot on the right, the top genes clearly standout by their distinct separation and by how they line up along the vertical axis. The top genes that the PM identified were genes identified in Steelman et al. [10]. In addition, it also identified genes that were not previously identified in their work.

A comparison of the PM and the CM is given in Table 2. In this table, the top 200 genes of the CM are selected as the base set. The number and percentage of the top 10, 20, . . ., 100 genes of the PM in this set are given. For an example, if one will to compute the percentage of top 10 genes found using the PM in comparison to the 200 genes found using CM (base set). The computation can be done simply dividing the number of genes in common between two groups by 10. This analysis is represented by the first three columns in the table. In addition, this table gives results that switch the roles of the PM and CM. More specifically, the top 200 genes of the PM are selected as the base set and the number and percentage of the top 10, 20, . . ., 100 genes of the CM in this set are determined. This analysis is represented by the last three columns in Table 2. With the CM as the base set, the results range from 70% of the top 10 genes to 22% of the top 100 genes of the PM being in set of the top 200 genes of the CM. Similarly, with the PM as the base set, the results range from 50% of the top 10 genes to 22% of the top 100 genes of the CM being in the set of the top 200 genes of the PM. Thus, while there is agreement between the two approaches, the lack of agreement warrants further investigation on the best choice of method based on the criteria of highest SP and lowest FDR. Our last study is a Monte Carlo simulation data study to compare these two approaches under these criteria.

**Table 2:** Top ranked genes of one method in the top 200 genes of the other method in study (ii)

x	x in top 200 CM genes	%x in Top 200 CM genes	y	y in top 200 PM genes	%y in Top 200 PM genes
**10**	7	70	**10**	5	50
**20**	9	45	**20**	7	35
**30**	10	33	**30**	7	23
**40**	10	25	**40**	11	28
**50**	11	22	**50**	15	30
**60**	17	28	**60**	17	28
**70**	19	27	**70**	17	24
**80**	20	25	**80**	19	24
**90**	20	22	**90**	20	22
**100**	21	21	**100**	21	21

This table shows how many of the top genes for one method are in the top 200 genes of the other method with × = # of top PM genes and y = # of top CM genes. For example, the result for × = 30 means that 10 (33%) of the top 30 genes of the PM where in the top 200 genes of the CM.

#### Simulation Study

As stated above, the purpose of the simulated data study is to evaluate and compare the PM and CM to identify genes with significant differential effects. We simulated several data sets based on the statistical properties of the data matrix from the second study. More specifically, each data matrix contained 40,000 genes with 10 assays of five samples in each group. The distribution for the simulated data can be described as follows:

(24) $$x_{ij}\overset{}{\sim }N\left(\mu_{x_{j}},\sigma_{x_{i}}^{2}\right),\;\forall i,j$$

such that

(25) $$\mu_{x_{j}}=\{\begin{array}{cc} \begin{array}{l} 5.3+\delta ,\\ 5.3, \end{array} & \begin{array}{l} \delta >0;i=1,\;\ldots ,\;200;\;j=1,\ldots ,5\\ otherwise \end{array} \end{array}$$

Thus, 200 of the genes for each of the assays in Group A had the largest mean and were significantly different than all the other genes that had a mean of 5.3. The study will evaluate the ability of the CM and PM to identify these 200 genes when the variance for all the data in the data matrix is the same (Part 1) and when the variance differs from gene to gene (Part 2). Each result in the simulation study is an average of five trials. For simplicity, all the results in this study will be based on eigengene (EG) principal components (PCs) as it gave strong separation of the groups.

#### Simulation Study -- Part 1

**Figure 5:**
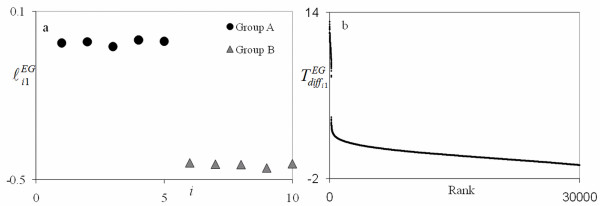
**Plot of Loading 1(a) and gene signature (b) when δ = 1 and σ = 0.20. Plot (a) shows a clean separation of Group A from Group B**. Plot (b) shows the nice assay-specific gene signature plotted against their rank.

**Figure 6:**
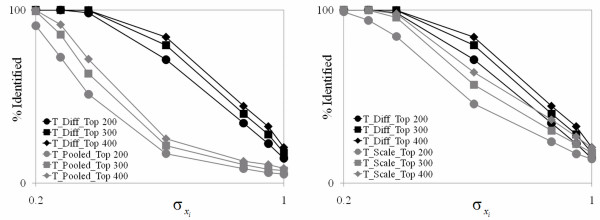
**Comparison of three test statistics in term of % of identified for simulation study**. This plot shows the percent of each method in accurately identified the 200 SDG. The plot of the left shows a comparison between $T_{diff}^{EG}$ and *T_pooled_*. While the plot of the right shows a comparison between $T_{diff}^{EG}$ and $T_{scaled}^{EG}$.

In the first simulation study we evaluated the techniques under different levels of $\sigma_{x}^{2}$ with δ = 1. (Note that for, δ = 1, the value of σ*_x _*is the same as the coefficient of variation defined as σ*_x_*/δ.) There were seven levels of $\sigma_{x}^{2}$ that ranged from 0.04 to 1.0. Thus, the range of the coefficient of variation was also 0.2 to 1.0. The PCA results for one trial of the PM at the lowest level of $\sigma_{x}^{2}$ are given in Figure 5. As shown, the loading plot indicates excellent separation of Group A and Group B indicating that PCA was able to pick up a difference of δ = 1 quite well for 200 of the 40,000 genes using the $T_{diff_{i1}}^{EA}$ test statistic. The signature plot reveals a distinct signature for these genes as evidenced by the large gap. For this case the percents of the 200 significantly different genes (SDG) ranked in the top 200 by $T_{diff_{i1}}^{EA}$, $T_{scaled_{i1}}^{EA}$ and $T_{pooled_{i}}^{EA}$, were 100.0%, 99.9% and 90.9%, respectively. These percentages for all the cases for this part of the simulation study for these three test statistics are given in Figure 6. In addition, this figure gives results for percentages of the SDG in the top 300 and top 400 for these test statistics. As shown, $T_{diff}^{EG}$ has the best performance, followed closely by $T_{scaled}^{EG}$ at the extremes and poorly by CM statistic *T_pooled_*. Thus, when the variability of the assays is similar, $T_{diff}^{EG}$ appears to be the best choice for identifying the most significant genes.

#### Simulation Study -- Part 2

In the second simulation study we evaluated the techniques by varying levels of $\sigma_{x_{i}}^{2}$ for each gene and two levels of δ: 1 and 3. More specifically, the distribution for $\sigma_{x_{i}}$ was log normal with mean 0.37 and variance 0.37^2^. Thus, for each data table a $\sigma_{x_{i}}$ was randomly generated for each gene *i*, *i *= 1, ..., *m*, and then ten simulated expression values, one for each assay, were generated according to Eqs. 24 and 25 for the given level of δ.

**Figure 7:**
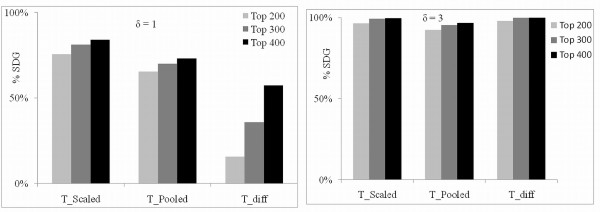
**The % of SDG in the top ranked genes for δ = 1 (left) and δ = 3 (right)**. This figure shows percent of the 200 significantly different genes (SDG) that are in top 200, top 300, and top 400 selected at two different values of δ for the *T_diff_*, *T_scaled_*, *T_pooled _*for Part 2 of the simulation study.

Identification results for this part of the study are given in Figure 7 as the percent of the SDG that are in the top 200 and top 400 ranks determined by the three test statistics. The best performing method this time is $T_{scaled}^{EG}$, followed by *T_pooled _*, and then by $T_{diff}^{EG}$. At δ = 3, all three methods are close but spread out at δ = 1. While the spread at δ = 1 for $T_{scaled}^{EG}$ and *T_pooled _*is significant, the spread for *T_pooled _*and $T_{diff}^{EG}$ is quite large. Thus, $T_{diff}^{EG}$ does not appear to be the best choice when δ is small and there is significant variation between the genes across the assays. Since $T_{scaled}^{EG}$ consistently did the best, when the gene variation is significant across the assays, it is our recommendation.

Our final analysis in this study evaluated performance in signature size determination. The CM is the Q-method developed by Storey and Tibshirani [12] that uses the p-values of the *t*-test (i.e., *T_pooled_*) and cuts the list off at a maximum Q-value, commonly 0.05, the value used in this analysis. The PM is the Inflection Method (IM) that is described in Rollins et al.[2] that cuts the list off at the greatest change in the signature plot of the ranked genes. The results are from Part 1 of the simulation study with a constant $\sigma_{x_{i}}$ for all the genes in a data table.

**Figure 8:**
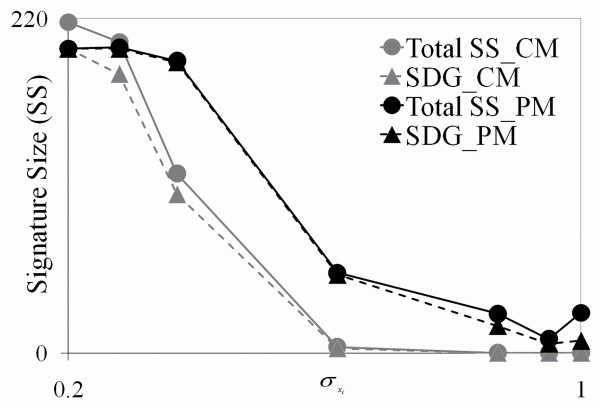
**Signature Size (SS) performance for the CM and PM**. The plot gives the signature sizes (i.e., number of genes the method determines as being significant) and the number of the 200 significantly different genes (SDG) in the signatures for the CM and PM. Higher statistical power (SP) is observed by the higher height of the SDG plot and the higher false discovery rate (FDR) is observed by the greater separation of the lines of the same color.

The results of this analysis are given in Figure 8. The plot gives the signature size (SS) (i.e., the number of genes in the signature) and the SDG against$\sigma_{x_{i}}$. Statistical Power (SP) is seen by the height of the SDG curve. As typical, SP, as indicated by this line, decreases as $\sigma_{x_{i}}$ increases. Hence, the PM signature performance is seen to be significantly better than the CM in terms of SP. An indication of the false discovery rate (FDR) of the methods can be compared by the separation of their two lines in Figure 8. These lines for the PM are very close except at the highest levels of$\sigma_{x_{i}}$. This indicates that the number of insignificant genes in the signatures of the PM is quite small and hence, has a small FDR. The FDR of the CM appears to be much higher for low values $\sigma_{x_{i}}$ and the SS drops to zero relatively quite fast so that performance at low $\sigma_{x_{i}}$ is not too meaningful since there are very few genes in the signature. Thus, the IM with the test statistics of the PM for determining signature cutoffs appears to have merit as a viable approach.

## Conclusion

This work proposed a new principal component analysis (PCA) method for analyzing large dimensional data set such as gene expressions data set. The strength of the proposed method (PM) comes from its data driven nature. It is data driven because the relationships obtained by PCA are only determined by those that exist in the data. Thus, no predetermined grouping or any á priori knowledge has influence on the principal components (PCs) obtained. After obtaining the PCs, they are used to match and verify the existence of the assay groups of interests. From the PCs that have the strongest match, the contribution of each gene providing the greatest differential expressions are identified and ranked. Thus, a PM signature is not just a difference of expression levels for genes but differences in a direction verified to have the characteristics of interests. This approach distinguishes PM from methods that do not form groups on the basis of data analysis and develop signatures from the differences between two groups in the original data space. One should be cautioned that as the number of members in the groups becomes smaller, the probability a particular order of the assays increases. Thus, for a small number of assays, one should require greater separation of groups for high confidence in the true existence of the groups.

Following Rollins et al. [2], the PM develops test statistics treating the assays as variables (eigengenes, EG) and the genes as variables (eigenassays, EA). These test statistics are linear combinations of these variables (i.e., pseudo variables) as determined from the elements of the eigenvectors. One test statistic, called *T_diff _*is the difference of the average expression levels between two groups of pseudo variables. The other test statistics, called *T_scaled_*, is *T_diff _*divided by the estimated pooled standard deviation. We compared the performance of these two test statistics with the common and popular Student's *t*-statistic, *T_pooled _*that we called the current method (CM). Two real data studies provided evidence in support of the PM as a viable technique. A simulation study provided the strongest supportive evidence for the use of *T_diff _*when the gene variability is fairly uniform throughout a data table and for *T_scaled _*when the variability is not fairly uniform. However, one should note that this study was done under a particular set of model assumptions. The most critical one is independence. If the data have a particular correlation structure, which is not uncommon given that all the genes in an assay experience the same set of conditions, the results in this article may not be supported. Future work will include evaluating the PM under the kinds of correlation structures found in real expression data. Finally, with the PM test statistics, the inflection method (IM) introduced by Rollins et al. [2], indicated strong promise in determining signature cutoffs in terms of statistical power and false discovery rate (FDR) as compared to CM.

We are applying the PM in a variety of applications involving biological as well as physical phenomenon, with promising results. These applications include: 1. Nitric Oxide- and S-nitrosoglutathione-responsive genes in *E*-*coli*; 2. analysis of DNA microarray data for juvenile small round blue cell tumors; 3. analysis of metabolite data from corn tissues (silk, pollen, coleoptile, and seedlings) for differential expression levels between the wild type and genetic mutations; 4. analysis of spectroscopy data for super alloys; and 5. the enhancement of nondestructive tests for ceramic armor in the resistance of ballistic penetration. Thus, the PM has potential application in a variety of situations where differential analysis is needed on large data sets with a relatively small number of different conditions or assays. It appears to have promise for these applications for high SP and low FDR as compared to other currently available methods.

## Abbreviations

CM: current method; EA: Eigenassay; EG: Eigengene; FDR: false discovery rate; FG: functional genomics; IM: inflection method; *g_i_*: gene contribution for *i^th ^*gene; *l*: loading; PCA: Principal Component Analysis; PC: Principal Component; PM: proposed method; *S*: score; SDG: statistically different genes; SP: statistical power; SS: signature size; *T_diff_*: difference of the average expression levels between two groups of pseudo variables; T_pooled_: Student's *t-*statistics; *T_scaled_*: scaled statistics by dividing *T_diff _*by its estimated pooled standard deviation; δ: differential effect

## Competing interests

The authors declare that they have no competing interests.

## Authors' contributions

DK developed and extended the methodology used in this work. DK and AL both participated in research to verify the methodology through a simulation study as well as real data studies, drafted the manuscript, read and approved the final manuscript.
